# The Skin in Health and Disease

**Published:** 1880-10

**Authors:** 


*The Skin in Health and Disease.*—By L. Duncan Bulkley, M. D. Attending Physician for Skin and Veneral Diseases at N. Y. Hospital, etc. Publisher: Presley Blakiston, Philadelphia, 1880.

This work is another of the series of the American Health Primers, and like its predecessors is a useful and interesting treatise. Its contents consist of the Anatomy and Physiology of the Skin, The Care of the Skin in Health, Diseases of the Skin, Diet and Hygiene in Diseases of the Skin. Much important information can be gleaned from the American Health Primers, as they are all prepared by competent authors.

